# Panhandle Medical Association

**Published:** 1900-09

**Authors:** 


### Panhandle Medical Association.

The Panhandle Medical Association, a new medical society, held its first meeting at Quanah, Texas, September 4 and 5, inst. Dr. David R. Fly is president and Dr. B. F. Hart is secretary. A large number of the Panhandle physicians attended, and an interesting program was carried out. We hope to be favored with a report of the meeting and some of the valuable papers read. It is to be regretted that in getting out the program the secretary omitted to give the postoffice address of the officers, and of those who contributed papers. We would be pleased to send a "sample copy" to such of them as are not subscribers of the "Red-Back."

